# Trait and state anxiety reduce the mere exposure effect

**DOI:** 10.3389/fpsyg.2015.00701

**Published:** 2015-05-28

**Authors:** Sandra L. Ladd, John D. E. Gabrieli

**Affiliations:** ^1^Department of Behavioral Neuroscience, Division of Graduate Medical Sciences, Boston University School of MedicineBoston, MA, USA; ^2^Department of Brain and Cognitive Sciences, Massachusetts Institute of TechnologyCambridge, MA, USA

**Keywords:** trait and state anxiety, affective processes, mere exposure effect, implicit memory, processing fluency

## Abstract

The mere exposure effect refers to an affective preference elicited by exposure to previously unfamiliar items. Although it is a well-established finding, its mechanism remains uncertain, with some positing that it reflects affective processes and others positing that it reflects perceptual or motor fluency with repeated items. Here we examined whether individual differences in trait and state anxiety, which have been associated with the experience of emotion, influence the mere exposure effect. Participants’ trait (Study 1) and state (Study 2) anxiety were characterized with the State-Trait Anxiety Inventory. Greater trait and state anxiety correlated with greater negative affect and lesser positive affect. In both experiments, greater anxiety was associated with a reduced mere exposure effect. Measures of fluency (response times at study and test) were unrelated to the mere exposure effect. These findings support the role of affective processes in the mere exposure effect, and offer a new insight into the nature of anxiety such that anxiety is associated with a reduced experience of positive affect typically associated with familiarity.

## Introduction

The “mere exposure effect” was introduced by Zajonc (1968) to describe a ubiquitous phenomenon first observed over a century ago (Fechner, 1876): unfamiliar items, when encountered again, elicit increased preference. Despite over four decades of extensive scientific inquiry spearheaded by his research, the psychological mechanism underlying the mere exposure effect remains uncertain (reviewed in Butler and Berry, 2004; Moreland and Topolinski, 2010). Two major explanatory frameworks for the mere exposure effect are (a) emotional or affective processing (Zajonc, 1968; Kunst-Wilson and Zajonc, 1980; Zebrowitz and Zhang, 2012; Ladd et al., 2014), and (b) processing fluency or ease of processing (Bornstein and D’Agostino, 1994; Seamon et al., 1995; Topolinski and Strack, 2009). Here we examined whether trait and state anxiety (Spielberger et al., 1983), which have been associated with the experience of emotion (Watson et al., 1988; Clark and Watson, 1991), independent from processing fluency, influence the mere exposure effect. Such an influence would support the view that the mere exposure effect is associated with affect as originally proposed (Zajonc, 1968), and provide an insight into the nature of anxiety.

Affective processing was originally posited to explain the mere exposure effect. Novel stimuli may evoke instinctive fear reactions that, in the absence of danger, decrease when exposed again (mere exposure); an increase in positive affect (higher liking ratings) results from the attenuation of fear (Zajonc, 1968). Cardiovascular and behavioral evidence suggests that the relaxation response is associated with the mere exposure effect. Specifically, peripheral vasodilatation during encoding predicted preference for studied nonwords during retrieval (Ladd et al., 2014). Vasodilatation, the reciprocal of decreased heart rate (Cowings and Toscano, 2000; Cowings et al., 2001, 2007), is a core component of the parasympathetic nervous system (PNS)-dominant pattern called the relaxation response (Benson, 1975, 1983; Benson et al., 1975).

Chronic anxiety (trait) is a relatively stable individual difference in the tendency to perceive stressful situations as dangerous or threatening; fluctuating anxiety (state) is this same tendency that varies over time (Spielberger, 1966). If the relaxation response, a specific form of positive affect (Steptoe et al., 2005), drives increased preference for familiar stimuli, then anxiety, the opposite condition to relaxation (Hofmann et al., 2005; Miu et al., 2009), may reduce the mere exposure effect. Anxiety, a prolonged mood, is also known to be associated with variation in acute emotional experiences because greater anxiety is consistently correlated with more negative and fewer positive experiences of emotion (reviewed in Elwood et al., 2012; Rossi and Pourtois, 2012), a correlational pattern we expected to replicate in the present study. We hypothesized, therefore, that greater anxiety, whether chronic (trait anxiety, Study 1) or fluctuating (state anxiety, Study 2), would be associated with a reduced preference for repeated novel nonwords (a reduced mere exposure effect); such a finding would support an affective mechanism underlying the mere exposure effect.

Processing fluency (Jacoby and Whitehouse, 1989) is an alternative, cognitive explanation for the mere exposure effect. It posits that increased reports of liking for familiar relative to unfamiliar items are not driven by affect but by stimulus features that are perceptually easier to process for repeated than novel items (perceptual fluency, Seamon et al., 1995). Expanding on this view, when there is minimal awareness of the relationship between ease of processing and perceptual features, the experience of fluency is misattributed to liking (perceptual fluency/misattribution, Bornstein and D’Agostino, 1992, 1994). This cognitive approach is also used to explain the increased preference observed with implicitly learned grammatical letter sequences (structural mere exposure effect, Gordon and Holyoak, 1983; Newell and Bright, 2001). In addition to variations on perceptual fluency, stimulus-specific motor reenactments or subvocalizations have been described as the mechanism underlying preference for repeated items (motor fluency, Topolinski and Strack, 2009; Topolinski, 2012).

In two studies, we examined whether affective or fluency measures were related to the mere exposure effect. The mere exposure effect was measured using the protocol that introduced the phenomenon into the psychological literature (Zajonc, 1968). The stimuli were pronounceable nonwords that were novel, meaningless, and carried no prior affective or semantic associations at encoding (study). In order to promote implicit, non-conscious processes, no reference was made as to any relation between the study phase, in which participants simply read the nonwords aloud, and the test phase, in which they selected which of two nonwords was preferred (with one repeated and one new nonword in each pair).

Anxiety and emotional experiences associated with anxiety were measured with widely used and well-validated self-report measures (reviewed in Spielberger et al., 1983; Watson et al., 1988; Kaplan and Saccuzzo, 1993; Mackinnon et al., 1999). Chronic and fluctuating forms of anxiety were measured with the State–Trait Anxiety Inventory (STAI–trait and state forms; Spielberger et al., 1983). In order to make a direct link between anxiety and emotional experience, we also administered the Positive and Negative Affect Schedule (PANAS–trait and state forms; Watson et al., 1988), a measure of emotionality.

We investigated processing fluency by measuring response times at study and at test. We measured speed of reading nonwords at study, and speed of preference judgments at test. We hypothesized that if processing fluency supported the mere exposure effect, then participants would read nonwords more quickly at study that would later be preferred than not preferred at test (i.e., greater reading fluency at study would be related to greater preference at test), and/or make preference judgments more quickly for repeated nonwords than novel nonwords (i.e., more fluent judgments at test would be related to preference for repeated items).

## Study 1

The goal of Study 1 was to examine the relationship between chronic anxiety (trait) and the mere exposure effect. In Study 1, each participant completed self-report trait anxiety (STAI–trait) and emotional experience (PANAS–trait) inventories prior to the administration of a computerized version of the mere exposure effect test. Voice response times during study and manual response times (mRTs) during test were the chronometric measures used to evaluate the relationship between processing fluency and preference.

### Materials and Methods

#### Participants

Nine men (age: *M* = 28.67 years, SD = 6.58) and 15 women (age: *M* = 24.67 years, SD = 4.99), selected from the general and student population in Boston, MA, USA, participated in this study that was conducted in the Clinical Research Center (CRC) on the Massachusetts Institute of Technology (MIT) campus. Participants volunteered after providing informed consent to a protocol approved by the Committee on the Use of Humans as Experimental Subjects (COUHES) and received monetary compensation for their participation.

#### Measures

##### State–Trait Anxiety Inventory (STAI–trait form; Spielberger et al., 1983)

The STAI–trait form was designed to assess relatively stable individual differences in the tendency to perceive stressful situations as dangerous or threatening. Participants responded to 20 self-report questions concerning how they generally feel on a 4-point Likert scale. The validity and reliability of the STAI–trait form has been established in over 3,300 studies, including research in medicine, dentistry, education, and the behavioral sciences (reviewed in Spielberger et al., 1983; Kaplan and Saccuzzo, 1993). Prior to conducting Pearson’s Product-Moment Correlations (Pearson’s correlations), trait anxiety scores were evaluated for outliers and for distribution normality. No outliers for trait anxiety scores were found and the distribution did not differ significantly from normal. All scores were within ±2.5 SDs of their respective Ms. In order to compare the distribution here with other studies where measurement was based on different scales, raw scores were converted to standardized scores (*z*). Total standardized trait anxiety scores were the data for this measure.

##### Positive and Negative Affect Schedule (PANAS–trait form; Watson et al., 1988)

In order to relate the chronic form of anxiety to chronic emotional experiences, we administered the PANAS. The PANAS–trait form was designed to assess two dominant, relatively independent, and stable dimensions of positive and negative emotionality. Participants respond to 20 words by listing a number from a 5-point scale next to each word that describes how they generally feel or feel on the average (e.g., 1-*very slightly or not at all*; 5-*extremely*). The PANAS–trait form, a widely used instrument, has good validity and reliability (Watson et al., 1988; Mackinnon et al., 1999). The negative subscale (NA) of the PANAS measures subjective distress and includes aversive emotional experiences such as anger, contempt, disgust, guilt, fear, and nervousness. Low NA indicates calm and serene feelings. The positive subscale (PA) of the PANAS measures subjective feelings of high energy, alertness, enthusiasm, and full concentration. Low PA indicates sad and lethargic feelings. Scores on the NA and PA subscales of the PANAS were evaluated for outliers and for distribution normality. No outliers for subscale scores were found and the distributions did not differ significantly from normal. All scores were within ±2.5 SDs of their respective Ms. Total scores on the NA and PA subscales (10 items each) of the PANAS–trait form were the data for this measure.

##### Mere exposure effect measure

The mere exposure effect test was administered using a Macintosh computer and PsychLab software, version 1.092. The PsychLab software presented stimuli, recorded response times in milliseconds (ms), and registered the right/left key inputs used to indicate preference. The stimuli were 48 pronounceable nonwords (Turkish words or pseudowords), all eight letters long (Zajonc, 1968). Half the nonwords were assigned to Study List A, and the remaining half were assigned to Study List B. For the test lists, nonwords from Study List A and B were paired. The position (right or left) was randomly assigned to the nonwords on the first test form and reversed on the second test form, with the constraint that half of the nonwords from each study list appeared on the left and the remaining half appeared on the right on each test form. The pairs were arranged in pseudorandom order with the constraint that no more than three items from the same study list appeared in the same location (right or left). For participants who studied List A (old items), List B was baseline (new items), and for participants who studied List B (old items), List A was baseline (new items). Thus, across participants, nonwords were counterbalanced as old or new, and the left/right positions of old and new nonwords were counterbalanced in each test form.

Scores for studied nonwords were evaluated for outliers and for distribution normality. No outliers for preference scores were found and the distribution did not differ significantly from normal. All scores were within ±2.5 SDs of their respective Ms. In order to compare the distribution here with other studies where measurement was based on different scales, raw scores were converted to standardized scores (*z*). Total standardized score for nonwords preferred was the data for this measure.

##### Processing fluency measures

Processing fluency was measured by comparing Ms of median stimulus-specific reading response times (vRTs) during study (encoding) and mRTs during test (retrieval). For the encoding phase, response times were compared for nonwords subsequently preferred relative to not preferred at test. For the retrieval phase, response times were compared for studied nonwords that were preferred relative to not preferred at test.

#### Procedure

Before the mere exposure effect test was administered, participants filled-out a self-report inventory for trait anxiety (STAI–trait form; Spielberger et al., 1983) and for emotional experience (PANAS–trait form; Watson et al., 1988). Five practice trials preceded both the study and test phases of the mere exposure effect test. At study, participants read 24 novel nonwords into a voice response relay. At test, 48 studied and unstudied nonwords were presented in 24 pairs and participants pressed the key directly in front of each nonword that they preferred. During the study phase, the subsequent test phase was not mentioned. Accordingly, no reference to the study list was made during the test phase. For the study phase, participants were told to read each nonword presented on the monitor as quickly and accurately as possible. Each trial began with a fixation cross presented for 500-ms followed by a 500-ms inter-stimulus interval (ISI) and then a nonword for 2,000-ms. The RTv for each trial was collected with a voice-activated relay connected to a computer. During the study phase, each item’s RTv initiated the next trial. For the test phase, each trial began with a fixation cross presented for 500-ms followed by a 500-ms ISI. Then, two nonwords were presented side by side. Participants were told that (a) each of their index fingers was to continually rest on the right (R) or left (L) key on the keyboard, (b) two paired nonwords would appear in the center of the monitor, and (c) their task was to press the key that was directly across from the nonword that they preferred (R or L key). On the keyboard, the “o” key was relabeled R and the “r” key was relabeled L so that participants pressed the key that was directly across from the preferred nonword.

### Results

#### Mere Exposure Effect

The mere exposure effect was obtained because participants preferred studied nonwords (*M* = 59.03, SD = 15.33) greater than chance, one-tailed one-sample *t*-test, *t*(23) = 2.89, *p* = 0.004, *d* = 0.59.

#### Trait Anxiety and Mere Exposure Effect

**FIGURE 1:**
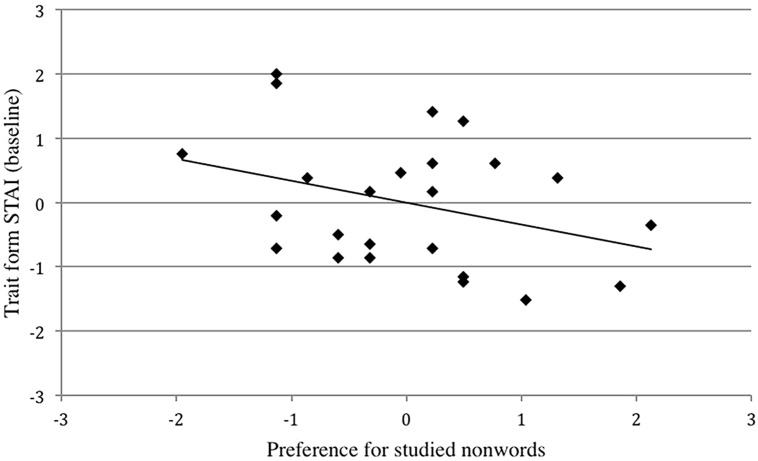
**Scatterplot of standardized-scores for the trait form of the State–Trait Anxiety Inventory (STAI) with preference for studied nonwords, Study 1.** Greater trait anxiety correlated with reduced preference for studied nonwords, *r* = -0.34, *p* = 0.050.

Raw scores were converted to *z* scores and a simple linear regression was conducted predicting preference for studied nonwords from baseline scores on the trait form of the STAI. Ms and SDs for raw data, from which the standardized-scores were derived, were computed for preferred studied nonwords (*N* = 24, *M* = 14.17, SD = 3.68) and baseline scores on the STAI–trait form (*N* = 24, *M* = 43.75, SD = 13.65). Baseline scores on the STAI–trait form were a significant negative predictor of preference scores for studied nonwords (*Beta* = -0.34, *p* = 0.049) indicating that higher trait anxiety was associated with lower preference scores for studied nonwords. As hypothesized, greater trait anxiety was associated with a diminished mere exposure effect (Figure 1).

#### STAI and PANAS –Trait Forms

To determine whether the PANAS, a measure of emotionality, was associated with anxiety as measured by the STAI, scores for the trait form of each self-report inventory were compared. Ms and SDs were computed for baseline scores on the STAI (*N* = 24, *M* = 43.75, SD = 13.65) and both subscales of the PANAS: NA (*N* = 24, *M* = 33.08, SD = 5.81) and PA (*N* = 24, *M* = 18.43, SD = 6.92). Greater trait anxiety (STAI scores) correlated significantly (*r* = 0.90, *p* < 0.001) with more negative emotional experience (higher NA subscale scores on the PANAS) and with lesser (*r* = -0.48, *p* = 0.009) positive emotional experience (lower PA subscale scores on the PANAS).

#### Processing Fluency and Preference

Response time analyses were used to examine the alternative hypothesis that test performance was a function of processing fluency during study (RTv) or test (RTm). Means of median RTv for studied nonwords that were subsequently preferred (*M* = 1328.6, SD = 415.1) compared to not preferred (*M* = 1367.8, SD = 508.9) at test did not significantly differ, one-tailed paired *t*-test, *t*(23) = 0.72, *p* = 0.239, *d* = 0.08. Means of median RTm for studied nonwords that were preferred (*M* = 1278.2, SD = 451.4) compared to not preferred (*M* = 1327.5, SD = 454.6) at test also did not significantly differ, one-tailed paired *t*-test, *t*(23) = 0.90, *p* = 0.187, *d* = 0.11.

## Study 2

Trait anxiety (e.g., chronic) reflects the history of state anxiety (e.g., transient) so that trait and state anxiety are highly correlated across a variety of groups and settings (e.g., Spielberger, 1966; Spielberger et al., 1983). In Study 1, greater trait anxiety was associated with a reduction in affective preference judgments for novel, repeated stimuli. Based on this finding, it is reasonable to expect that a similar pattern would be observed for state anxiety, fluctuating amounts of anxiety that vary on a temporal basis.

The goal of Study 2 was to examine the relationship between state anxiety and the mere exposure effect using a design that allowed for both an experimental and a correlational approach. Although the experimental manipulation of more and less cognitively demanding task was ineffective in altering state anxiety (Supplementary Materials, Study 2 — experimental approach) which precluded testing the experimental hypothesis that high state anxiety would produce a greater reduction in the mere exposure effect relative to low state anxiety, the correlational hypothesis that state anxiety would be associated with a diminished mere exposure effect remained testable. In Study 2 (i.e., correlational approach), each participant filled-out a self-report inventory for state anxiety (STAI–state form; Spielberger et al., 1983) and for emotional experience (PANAS–state form; Watson et al., 1988) both before and after the administration of the same mere exposure effect test used in Study 1. We used the means of the inventories before and after test performance as the best estimates of anxiety and emotionality during the mere exposure effect test.

### Materials and Methods

#### Participants

Eighteen men (age: *M* = 24.72 years, SD = 4.07) and 30 women (age: *M* = 23.93 years, SD = 4.71), selected from the general and student population in Boston, Massachusetts, participated in this study that was conducted in the CRC on the MIT campus. Participant volunteers provided informed consent to a protocol approved by COUHES and received monetary compensation for their participation. None of the participants in Study 2 were selected from the sample used in Study 1.

#### Measures

##### State–Trait Anxiety Inventory (STAI–state form; Spielberger et al., 1983)

The STAI–state form was designed to assess individual differences that fluctuate over time in the tendency to perceive stressful situations as dangerous or threatening. Participants respond to 20 self-report questions concerning how they felt in the present moment on a 4-point Likert scale. The STAI–state form has strong validity, but was not expected to have strong reliability because this form of anxiety is expected to fluctuate over time (reviewed in Spielberger et al., 1983; Kaplan and Saccuzzo, 1993). Prior to conducting Pearson’s correlations, state anxiety scores (Ms for baseline and post-test) were evaluated for outliers and for distribution normality. Data points more than ± 2.5 SDs from the *M* were considered outliers and there were three baseline-STAI and two post-STAI. The five participants reporting outlier scores were removed from the study because outlier values can greatly influence correlations. All statistical analyses were conducted on the remaining 43 participants. To facilitate comparisons between the distributions here with studies using different scales, raw scores were converted to *z* scores. Total standardized state anxiety scores were the data for this measure.

##### Positive and Negative Affect Schedule (PANAS–state form; Watson et al., 1988)

In order to relate the fluctuating form of anxiety to fluctuating emotional experiences, we administered the PANAS. The PANAS–state form was designed to assess two dominant and relatively independent dimensions of positive and negative emotionality. Participants respond to 20 words that describe different emotional experiences by listing a number from a 5-point scale next to each word that describes how they feel right now (e.g., 1-*very slightly or not at all*; 5-*extremely*). The PANAS–state form, has good validity, but was not expected to have strong reliability because this form of emotional experience is expected to fluctuate over time (Watson et al., 1988; Mackinnon et al., 1999). The descriptions of the NA and PA subscales of the PANAS–state form are the same as those used for the PANAS–trait form; emotional experiences do not differ in kind, but in frequency. Scores on the NA and PA subscales (10 items each) of the PANAS were evaluated for outliers and for distribution normality. No outliers were found and the distributions did not differ significantly from normal. All scores were within ±2.5 SDs of their respective Ms. Total scores on the NA and PA subscales (10 items each) of the PANAS–state form were the data for this measure.

##### Mere exposure effect measure

This measure was the same as that used in Study 1. No outliers for preference scores were found and the distribution did not differ significantly from normal. All scores were within ±2.5 SDs of their respective Ms. Total standardized score for nonwords preferred was the data for this measure.

##### Processing fluency measures

These measures were the same as those used in Study 1.

#### Procedure

Each participant was given the mere exposure effect test using the same instructions, hardware, and software as that described in Study 1. Before the mere exposure effect test was administered, participants filled-out the STAI–state form (Spielberger et al., 1983) and the PANAS–state form (Watson et al., 1988). After the mere exposure effect test was administered, participants filled-out these self-report inventories again, because state anxiety is expected to fluctuate over time.

### Results

#### Mere Exposure Effect

The mere exposure effect was obtained because participants preferred studied nonwords (*M* = 58.14, SD = 14.38) greater than chance, one-tailed one-sample *t*-test, *t*(42) = 3.71, *p* = 0.0003, *d* = 0.57.

#### State Anxiety and Mere Exposure Effect

**FIGURE 2:**
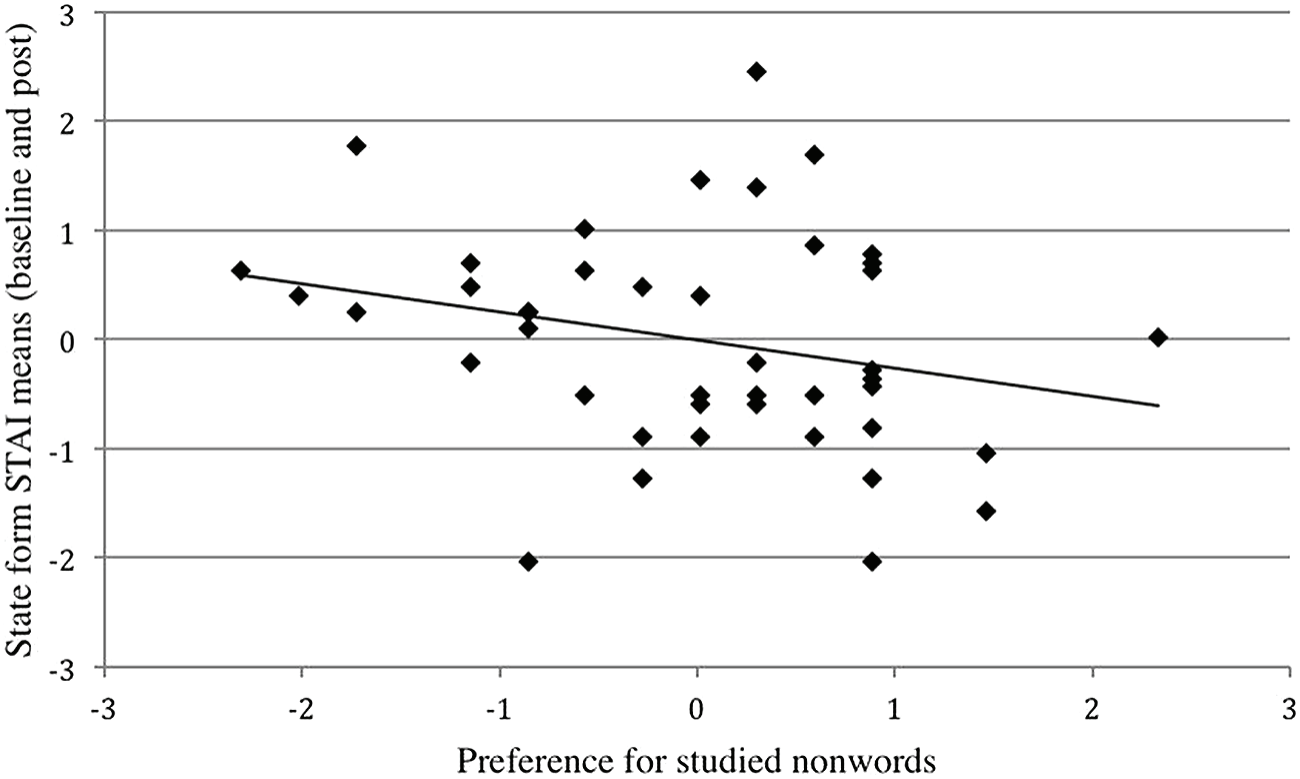
**Scatterplot of standardized-scores for the state form of the STAI with preference for studied nonwords, Study 2.** Greater state anxiety correlated with reduced preference for studied nonwords, *r* = -0.26, *p* = 0.047.

Raw scores were converted to *z* scores and a simple linear regression was conducted predicting preference for studied nonwords from mean scores on the state form of the STAI. Ms and SDs for raw data from which the standardized-scores were derived were computed for preferred studied nonwords (*N* = 43, *M* = 13.95, SD = 3.45) and mean scores (baseline and post-test) on the STAI–state form (*N* = 43, *M* = 33.35, SD = 6.75). Mean scores on the STAI–state form were a significant negative predictor of preference scores for studied nonwords (*Beta* = -0.26, *p* = 0.047) indicating that higher state anxiety was associated with lower preference scores for studied nonwords. As hypothesized, greater state anxiety was associated with a diminished mere exposure effect (Figure 2).

Ms and SDs for raw data from which the standardized-scores were derived were also computed separately for baseline (*N* = 43, *M* = 33.42, SD = 6.93) and post-test (*N* = 43, *M* = 33.28, SD = 8.74) scores on the STAI–state form. A significant one-tailed negative correlation was observed between mean baseline scores on the STAI–state form and preference for studied nonwords, *r* = -0.42, *p* = 0.002. A parallel finding was not observed when the same analysis was computed for the post-test scores on the STAI–state form, *r* = -0.052, *ns*. State anxiety is defined as fluctuating over time. A discrepancy between baseline and post-test scores would be the expected finding and represents the rationale for using the mean (baseline and post-test).

#### STAI and PANAS – State Forms

To determine whether the PANAS, a measure of emotionality, was associated with anxiety as measured by the STAI, scores for the state form of each self-report inventory were compared. Ms and SDs were computed for mean scores (baseline and post-test) on the STAI (*N* = 43, *M* = 33.35, SD = 6.57) and both subscales of the PANAS: NA (*N* = 43, *M* = 12.59, SD = 2.30) and PA (*N* = 43, *M* = 28.13, SD = 7.42). Greater state anxiety (STAI scores) correlated significantly (*r* = 0.51, *p* < 0.001) with more negative emotional experiences (higher NA subscale scores on the PANAS) and with lesser (*r* = -0.37, *p* = 0.007) positive emotional experiences (lower PA subscale scores on the PANAS).

#### Processing Fluency and Preference

Response time analyses were used to examine the alternative hypothesis that test performance was a function of processing fluency during study (RTv) or test (RTm). Means of median RTv for studied nonwords that were subsequently preferred (*M* = 1247.2, SD = 421.2) compared to not preferred (*M* = 1254.9, SD = 376.7) at test did not significantly differ, one-tailed paired *t*-test, *t*(42) = 0.39, *p* = 0.349, *d* = 0.02. Means of median RTm for studied nonwords that were preferred (*M* = 1249.5, SD = 386.4) compared to not preferred (*M* = 1264.4, SD = 423.3) at test, also, did not significantly differ, one-tailed paired *t*-test, *t*(42) = 0.71, *p* = 0.242, *d* = 0.04.

## Discussion

Greater anxiety, whether chronic or fluctuating, was associated with a reduced mere exposure effect. Higher STAI scores in both studies correlated significantly with lower mere exposure effects. Anxiety was associated with a pattern of emotional experiences. Greater trait and state anxiety were both correlated with more frequent negative and less frequent positive affect (as measured with the PANAS). Perceptual fluency, measured as response times at study and at test, was unrelated to the mere exposure effect. These findings are consistent with the affective explanation for the mere exposure effect originally posited by Zajonc (1968) and, at the same time, offer a new insight into the nature of anxiety such that anxiety may reduce the experience of positive affect that is typically associated with familiarity.

The observation that the mere exposure effect was diminished, at approximately equivalent levels, by both trait and state anxiety is consistent with the observation that increased vasodilatation, as measured by blood volume pulse during encoding, predicts affective preference during retrieval (Ladd et al., 2014). Vasodilatation is the relative reciprocal of heart rate (Cowings et al., 2001). Decreased heart rate is a core component of a PNS–dominant pattern referred to as the relaxation response (Benson, 1975, 1983; Benson et al., 1975). PNS versus sympathetic-dominant autonomic patterns have been incorporated into contemporary theories on emotion (Kreibig et al., 2007; Sequeira et al., 2009; Kreibig, 2010). Because anxiety and the relaxation response represent incompatible patterns of affective responding (Hofmann et al., 2005; Miu et al., 2009), the finding that greater anxiety, in both its sustained (trait) and transient (state) form, is associated with reduced preference for studied nonwords provides converging evidence for the suggestion that the relaxation response may drive the mere exposure effect.

The findings that variation in anxiety and related emotional experience were associated with the magnitude of the mere exposure effect, but that measures of fluency were not, aligns with clinical evidence that the mere exposure effect is compromised in affective but not cognitive disorders. Patients with the affective disorder of depression have been reported to have an absence of a mere exposure effect (Quoniam et al., 2003). In contrast, patients with disorders that are primarily cognitive or perceptual in nature have exhibited intact mere exposure effects, including patients with Alzheimer’s disease (Quoniam et al., 2003), schizophrenia (Marie et al., 2001), amnesia in alcoholic Korsakoff’s syndrome (Johnson et al., 1985), transient global amnesia (Marin-Garcia et al., 2013), and prosopagnosia (Greve and Bauer, 1990). Although alternative explanations for a reduced mere exposure effect in depression are possible, such as greater distractibility (Lemelin et al., 1997), it is striking that two variations of negative emotional experience, depression and anxiety, both diminished the mere exposure effect.

In contrast to the findings reported here for trait anxiety, both null results and those in the opposite direction have been observed in two research reports that investigated similar hypotheses but used procedures that encouraged conscious preference formation (Schick et al., 1972; Campbell and McKeen, 2011). A possible relationship between intentionality and the nature of preference formation may explain this apparent inconsistency. The prior studies used meaningful stimuli (faces, Campbell and McKeen, 2011; cartoon characters, Schick et al., 1972) and gave explicit information concerning the relationship between study and test (personal approachability judgments based on prior exposure/appearance, Campbell and McKeen, 2011; affective judgment booklets in which pages contained both study and test stimuli, Schick et al., 1972). Accordingly, other investigators proposing that the mere exposure effect can be explained by processing fluency, rather than affect, have used procedures that overlap encoding and retrieval performance demands (e.g., requiring liking ratings at both study and test) or instructions that connect study to test (e.g., describing the experiment as a memory test which implies stimulus repetition). This methodology encourages awareness of the relationship between study and test stimuli and the results generated from it may be most informative when describing conscious preference formation (Hupbach et al., 2006, Experiments 1 and 2; Lawson, 2004, Experiment 1; Topolinski and Strack, 2009, Experiments 1–3).

One limitation of the present investigation was that in Study 2 an experimental manipulation involving cognitive tasks was meant to increase state anxiety but failed to do so (Supplementary Materials, Study 2 — experimental approach). Because state anxiety was unaffected by the cognitive tasks that intervened between baseline and post-test measures, we could still examine the relation of state anxiety to the mere exposure effect. The state anxiety findings (Study 2) converged with the trait anxiety findings (Study 1). A future study that successfully manipulates anxiety, perhaps by using a method that has proven to provoke anxiety such as anticipated public speaking (e.g., Hofmann et al., 2005), could add a causal finding to the present correlational results.

Another limitation pertains to the measurement of fluency, which appeared unrelated to the mere exposure effect in both studies. We used response-time measures, and other investigators of processing fluency have used either manipulations of test-phase perceptual quality (e.g., Alter and Oppenheimer, 2009) or interference with stimulus-specific motor or effector processes (Topolinski and Strack, 2009). Also, our observation that speed of performance was unrelated to the mere exposure effect was a null finding, which is inherently weaker than the positive findings relating anxiety to the mere exposure effect.

Other lines of evidence, however, indicate that the mere exposure effect as measured here is unlikely to represent a perceptual and non-affective process. First, preference has not been seen when the affective nature of the question is reversed (i.e., which stimulus is disliked or not preferred; Zajonc, 1968; Zajonc et al., 1972, 1974a,b; Seamon et al., 1998). Second, as participants become aware of their misattributions, they correct for them (Mitchell et al., 2005), but the mere exposure effect is not diminished when the awareness of fluency increases and participants have the opportunity to correct for their misattribution of fluency to liking (e.g., Krugman and Hartley, 1960; Moreland and Zajonc, 1976; Seamon et al., 1984; Bornstein et al., 1987; Study 2).

The finding that anxiety, which correlated with negative and positive experiences of emotion, was related to the mere exposure effect, but that fluency appeared unrelated must be considered in the context of the specific experimental paradigm employed in the present study. The paradigm was similar to that from Zajonc (1968) in two main regards. First, the non-sense word stimuli were novel and meaningless and carried no prior affective or semantic associations. Second, the instructions promoted implicit or non-conscious memory processes during retrieval (at test) by making no reference to the test phase at study or the study phase at test. Indeed, in such designs, mere exposure effects are more pronounced when obtained under subliminal conditions than when participants are aware of the repeated exposures (Bornstein and D’Agostino, 1992; Murphy et al., 1995) and these subliminal exposure effects are diffuse since participants rate their own mood more positively after repeated exposures (Monahan et al., 2000), findings that have not been reported for other measures of implicit priming. Also, for verbal stimuli, unlike perceptual identification implicit priming, the mere exposure effect can only be reliably produced with nonwords (Butler et al., 2004). Thus, our findings that related anxiety, and associated emotional experiences, to the mere exposure effect occurred under conditions that maximized non-conscious or implicit processes in preference formation as measured by the mere exposure effect.

Together, the results reported here (Study 1 and 2) suggest that anxiety may reduce the experience of positive affect typically associated with familiarity. People with greater trait and state anxiety failed to exhibit the gains in positive affect for repeated items that were exhibited by people with lesser trait and state anxiety. It may be that one aspect of anxiety is the failure to find positive affect in the repeated experiences that are pervasive in our lives.

## Conflict of Interest Statement

The authors declare that the research was conducted in the absence of any commercial or financial relationships that could be construed as a potential conflict of interest.
