# Supplementary material for: Trait and state anxiety reduce the mere exposure effect
Source: Front Psychol. 2015 May 28;6:701. doi: 10.3389/fpsyg.2015.00701 (PMC4446535; doi:10.3389/fpsyg.2015.00701)
Supplement: Supplementary file 1 [file Data_Sheet_1.DOC]

***Supplementary Material***

**Induction of state anxiety and the mere exposure effect**

**Study 2 — Experimental approach**

**Sandra L. Ladd^1,2^* and John D. E. Gabrieli^2,1^**

^1^Boston University School of Medicine, Department of Behavioral Neuroscience, Division of Graduate Medical Sciences, Boston, MA, USA

^2^Massachusetts Institute of Technology, Department of Brain and Cognitive Sciences, Cambridge, MA, USA

***Correspondence:** Dr. Sandra Ladd, Massachusetts Institute of Technology, Department of Brain and Cognitive Sciences, Building 46, 43 Vassar Street, Cambridge, MA, 02139, USA. sladd@mit.edu

**Abstract**

The influence of state anxiety on the mere exposure effect (affective preference for novel items upon repetition) was investigated using an experimental approach. Here we examined whether high and low state anxiety, induced by completing high and low cognitive demanding tasks, influenced the mere exposure effect. In a preliminary study a high cognitive demanding task associated with the executive function of cognitive-switching (a measure of cognitive flexibility) was developed and compared with a low cognitive demanding task associated with interference (Stroop). The task-based hypothesis was supported: performance on cognitive-switching (high cognitive demand), relative to interference (low cognitive demand), produced significantly greater errors and longer response times overall and by condition. However, the experimental hypothesis that high, relative to low, state anxiety would reduce the mere exposure effect could not be tested because the manipulation of cognitive demand associated with an executive function (cognitive-switching) did not influence state anxiety.

**Introduction**

Inducing state anxiety in the laboratory has been accomplished by tasks that differ in cognitive demand but without specificity in regards to the level and type of cognitive demand and associated neuroanatomical substrates (reviewed in Rossi and Pourtois, 2013). Cognitive functions at the high demand level have been broken down into specific components that include the regulation and control of working memory, abstract thinking, task flexibility, problem solving, planning, and execution. These cognitive processes are collectively referred to as executive functions and are mediated primarily by the prefrontal areas of the frontal lobe (Lezak et al., 2012). Although what combination of demand level and cognitive function is necessary and sufficient to evoke state anxiety has not been specified in the psychological literature, increasing the level of cognitive demand, in general, is expected to increase the level of state anxiety. There is considerable evidence, both behavioral and psychophysiological, that state anxiety is typically low when a task is easy and increases as a task becomes more difficult (O'Neil et al., 1966; Sieber et al., 1977; Eysenck, 1982; Zeidner, 1998).

The goal of Study 2 **—** experimental approach was (a) to induce high state anxiety by requiring completion of a cognitive task associated with a specific executive function, flexibility (Lezak et al., 2012), and, then, (b) test the influence of high, relative to low, state anxiety induced by high, relative to low, cognitive demand on the mere exposure effect. The high cognitive demand of flexibility was measured by tasks that required cognitive-switching. Such tasks were designed to assess an individual’s ability to abandon a previous response in order to generate a novel one in the presence of unanticipated environmental cues (Delis et al., 2001a, 2001b). The low cognitive demand of interference was measured by interference (Stroop, 1935).

In Study 2 — experimental approach, each participant filled-out the State–Trait Anxiety Inventory (STAI–state form; Spielberger et al., 1983) and the Positive and Negative Affect Schedule (PANAS–state form; Watson et al., 1988) both before and after the administration of either the high or low cognitive demanding task and the mere exposure effect test. The mean (baseline and post-test) was used as the best estimate of state anxiety during the mere exposure effect test. The association between state anxiety and emotional experience was examined by comparing mean scores on both inventories. Based on the findings in Study 1 and the evidence that trait anxiety reflects the history of state anxiety (e.g., Spielberger, 1966), it was hypothesized that greater state anxiety (high cognitive demand induced by cognitive-switching; Ladd, 2014) would produce a diminished mere exposure effect relative to lesser state anxiety (low cognitive demand induced by interference; Stroop, 1935).

**Materials and Methods**

**Participants**

The participants were the same as those used in Study 2 (i.e., correlational approach). None of the participants in Study 2 — experimental approach were selected from the sample used in Study 1.

**Measures**

***State–Trait Anxiety Inventory (STAI—state form; Spielberger et al., 1983)*.** The STAI—state form and participant inclusion criteria were the same as Study 2 (i.e., correlational approach).

***Positive and Negative Affect Schedule (PANAS–state form; Watson et al., 1988).*** The PANAS—state form, used to make a direct link between anxiety and emotional experience (Clark et al., 1991), and participant inclusion criteria were the same as Study 2 (i.e., correlational approach).

***High cognitive demanding task.*** Designing a high cognitive demanding task was accomplished in three stages (Ladd, 2014). First, an executive function test normally administered using a paper-and-pencil format in a clinical setting was revised for an experimental protocol by programming software for computer administration that incorporated reading response times and increasing the number of cognitive-switching conditions. The executive function test selected for this purpose was the Color-Word Interference Test (CWIT) that is included in the Delis—Kaplan Executive Function System (D—KEFS; Delis et al., 2001a, 2001b). Computerization and revision of the CWIT for use in an experimental protocol (e.g., CWITexp) is consistent with the purpose of the D–KEFS: "In addition to its clinical uses, the D–KEFS was also developed to assist researchers in charting the neural substrates of the highest forms of human cognition" (Delis et al., 2001a, p. 15). Second, the CWITexp was used to construct a high cognitive demanding task (HCD). The HCD contained the single cognitive-switching condition in the original CWIT and three new cognitive-switching conditions. The CWITexp was also used to construct a low cognitive demanding task (LDC), which was a modified version of Stroop interference and did not contain a cognitive-switching condition. Third, a preliminary study was run on 12 men (*M* = 24.42 years, *SD* = 5.90) and 12 women (*M* = 25.07 years, *SD* = 5.66), lower division community college students who participated in the study to fulfill a general psychology course requirement. The purpose of this preliminary study was to test the validity of the HCD and LCD tasks. The data used to measure task difficulty was the sum of errors and mean of median reading response times. Consistent with the process approach (Kaplan, 1990), error data was inclusive (no outliers were removed) and median response times were measured by the sum (rather than the mean). The hypothesis that HCD, relative to the LCD, would produce significantly greater errors and longer response times overall and by condition was supported.

***Mere exposure effect.*** This measure was the same as that used in Study 1 and Study 2 (i.e., correlational approach).

***Processing fluency measures.*** These measures were the same as those used in Study 1 and Study 2 (i.e., correlational approach).

**Procedure**

After the STAI–state form (Spielberger et al., 1983) and PANAS–state form (Watson et al., 1988) were administered, participants were assigned to either the HCD or LCD group so that the STAI–state anxiety baseline mean did not significantly differ between these groups. Participants completed either the HCD or LCD tasks followed by the mere exposure effect test that used the same instructions, hardware, and software as that described in Study 1 and in Study 2 (i.e., correlational approach). Following the completion of the mere exposure effect test, both self-report inventories were administered again.

**Results**

**STAI and PANAS – state forms**

Pearson’s Product-Moment Correlations betweens the state form of the STAI and PANAS were the same as those reported in Study 2 (i.e., correlational approach), indicating that greater state anxiety was associated with more negative emotional experiences and with lesser positive emotional experiences.

**Modulation of state anxiety in the laboratory**

In order to determine whether HCD and LCD tasks induced high and low levels of state anxiety, respectively, a mixed-variable ANOVA with cognitive demand and time of testing was computed for the state anxiety measure. Results for STAI revealed no effect of cognitive demand (HCD, LCD), time of testing (baseline vs. post-test), or interaction between cognitive demand and time of testing, *F* < 1.0. The expectation that HCD and LCD tasks would induce different levels of state anxiety was not supported, because a significant interaction was not observed between time of testing (baseline, post-test) and level of cognitive demand (HCD, LCD).

**Mere exposure effect**

The mere exposure effect for combined data (HCD and LCD groups) was the same as that reported in Study 2 (i.e., correlational approach). An analysis of the mere exposure effect for each group individually revealed that participants in HCD (*M* = 57.39, *SD* = 13.73) and LCD (*M* = 58.93, *SD* = 15.33) groups both preferred studied nonwords greater than chance, one-tailed 1-sample analyses, *t*(21) = 2.52, *p* = 0.009, *d* = 0.54, and *t*(20) = 2.67, *p* = 0.007, *d* = 0.58, respectively.

**Discussion**

The experimental hypothesis that greater state anxiety (high cognitive demand induced by cognitive-switching) would produce a diminished mere exposure effect relative to lesser state anxiety (low cognitive demand induced by interference) could not be tested because the manipulation of cognitive demand did not affect state anxiety. One explanation for this null finding with respect to the manipulation of state anxiety in the laboratory is that there may have been a difference in external validity between the high cognitive demanding task and the conditions comprising it. The overall task performance for the students from MIT and surrounding universities was parallel to that observed for the independent sample of lower division community college students. However, when task performance was analyzed by condition, the sample of students from MIT and surrounding universities revealed a learning effect, similar to one-trial learning, between the first and second cognitive-switching conditions. This rapid learning effect (a sharp decrease in errors) in the middle of the task, although not statistically significant, may have eliminated any influence of subsequent condition difficulty on state anxiety. In contrast, for the participants in the independent sample of lower-division community college students, there was no evidence of a learning effect after completing the first cognitive-switching condition. This analysis suggests that cognitive demand may be viewed as the interaction between task properties and participant characteristics, rather than simply as a function of either task properties or participant characteristics. These findings also suggest that assessing the external validity of a task by condition, an option available with a stratified design, provides a more meaningful estimate of external validity than only considering a single overall task score.

Furthermore, a comparison of the pattern of errors and latencies for the two different samples revealed that the slower response times observed as the cognitive-switching conditions progressed did not reflect differences in difficulty, but were, most likely, a measure of fatigue. Participants from MIT and surrounding universities exhibited a decrease in errors during the second cognitive-switching condition that occurred without a parallel decrease in response times. The pattern of longer latencies across conditions was almost identical to that observed for the independent sample of lower division community college students whose mean error rate continued to accelerate across cognitive-switching conditions.

Another explanation for the failure of task difficulty associated with cognitive-switching to affect state anxiety may be considered in the context of a subsequent report indicating that tasks with higher cognitive load require cognitive strategies that reallocate resources toward the task demand, thereby reducing state anxiety (Vytal et al., 2012). Based on this finding, the interaction between working memory, cognitive load, and state anxiety was examined using an exemplary methodology(Vytal et al., 2013). Verbal and spatial working memory tasks were presented at three discrete levels that progressed in difficulty as measured by cognitive load (n-back tasks of increasing difficulty; 1, 2, and 3-back). State anxiety was induced by threat of shock. Both objective (startle reflex, eye blink) and subjective (STAI–state form) methods were used to assess the effectiveness of this anxiety-induction. Test performance was compared across unsafe (threat of shock) and safe (no threat of shock) trials on tasks that differed in working memory test modality (verbal, visual) and cognitive load (low, medium, high). State anxiety impaired verbal working memory for low and medium, but not for high levels of cognitive load. In contrast, spatial working memory was not affected by either state anxiety or cognitive load. These results suggest that a complex relationship exists between state anxiety and tasks that measure the executive functions.

**References**

Clark, L. A., and Watson, D. (1991). Tripartitemodel of anxiety and depression:

Psychometric evidence and taxonomic implications. *J. Abnorm. Psychol*. 100,

416-336. doi:10.1037/0021-843X.100.3.316

Delis, D. C., Kaplan, E., and Kramer, J. H. (2001a). *Delis—Kaplan Executive Function System Examiner's Manual*. San Antonio, TX: The Psychological Corporation.

Delis, D. C., Kaplan, E., and Kramer, J. H. (2001b). *Delis—Kaplan Executive Function System Technical Manual*. San Antonio, TX: The Psychological Corporation.

Eysenck, M. W. (1982). *Attention and Arousal: Cognition and Performance*. Berlin, Germany: Springer-Verlag.

Kaplan, E. (1990). The process approach to neuropsychological assessment of psychiatric

patients. *J. Neuropsychiatry Clin. Neurosci.* 2, 72-87. Retrieved from<http://neuro.psychiatryonline.org.ezproxy.bu.edu/data/Journals/NP/3786/72.pdf>

Ladd, Sandra. *“Cardiovascular psychophysiological and behavioral evidence for an affective implicit priming mechanism* (PhD diss., Boston University School of

Medicine, Boston University, 2014). Retrieved from Dissertations and Thesis database. (UMI No. 3645432).

Lezak, M. D., Howieson, D. B., Bigler, E. D., and Tranel, D. (2012). *Neuropsychological Assessment*, 5^th^ edn. New York, NY: Oxford University Press.

O'Neil, H. F., Spielberger, C. D., and Hansen, D. N. (1969). The effects of state anxiety and task difficulty on computer-assisted learning. *J. Ed. Psychol.* 60, 343-350.

doi:10.1037/h0028323

Rossi, V. and Pourtois, G. (2012). Transient state–dependent fluctuations in anxiety measured using STAI, POMS, PANAS or VAS: A comparative review. *Anxiety Stress Coping* 25, 603-45. doi:10.1080/10615806.2011.582948

Sieber, J. E., O'Neil, H. F., and Tobias, S. (1977). *Anxiety, learning, and instruction*.

Hillsdale, NJ: Erlbaum.

Stroop, J. R. (1935). Studies of interference in serial verbal reactions. *J. Exp. Psychol*. 18,

643-662. Retrieved from<http://psychcentral.com/classics/Stroop/>

Spielberger, C. (1966). “Theory and research on anxiety,” in *Anxiety and Behavior*. ed.

C. D. Spielberger (New York, NY: Academic Press), 3–19.

Spielberger, C., Gorsuch, R., Lushene, R. E., Vagg, P., and Jacobs, G. A. (1983). *Manual*

*for the State–Trait Anxiety Inventory STAI (Form Y)*. Palo Alto, CA: Consulting Psychologist Press.

Vytal, K., Cornwell, B., Arkin, N., and Grillon, C. (2012). Describing the interplay between anxiety and cognition: from impaired performance under low cognitive load to reduced anxiety under high load. *Psychophysiology* 49, 842-852. doi:10.1111/j.1469-8986.2012.01358.x

Vytal, K. E., Cornwell, B. R., Letkiewicz, A. M., Arkin, N. E., and Grillon, C. (2013).

The complex interaction between anxiety and cognition: Insight from spatial and verbal working memory. *Front. Hum. Neurosci.* 7, 1-11. doi:10.3389/fnhum.2013.00093

Watson, D., Clark, L. A., and Tellegen, A. (1988). Development and validation of brief

measures of positive and negative affect: the PANAS scales. *J. Pers. Soc.*

*Psychol*. 54, 1063-1070. doi:10.1037/0022-3514.54.6.1063

Zeidner, M. (1998). *Test Anxiety: The State of the Art*. New York, NY: Plenum Press.
